# Metal- and polymer-assisted SEM imaging of protein-bound DNA molecules: a protocol

**DOI:** 10.1093/biomethods/bpag052

**Published:** 2026-09-04

**Authors:** Chanyoung Noh, Taebin Yun, Minseo Kang, Kyubong Jo

**Affiliations:** Department of Chemistry, Sogang University, 35 Baekbeom-ro, Mapo-gu, Seoul 04107, Korea; Department of Chemistry, Sogang University, 35 Baekbeom-ro, Mapo-gu, Seoul 04107, Korea; Department of Chemistry, Sogang University, 35 Baekbeom-ro, Mapo-gu, Seoul 04107, Korea; Department of Chemistry, Sogang University, 35 Baekbeom-ro, Mapo-gu, Seoul 04107, Korea

**Keywords:** scanning electron microscopy, single-molecule DNA imaging, protein–DNA interactions, polymer-assisted imaging, DNA–protein complexes

## Abstract

Direct mapping of protein positions along long, stretched DNA molecules requires simultaneous visualization of the DNA backbone and associated protein signals. We recently established a heavy-metal- and polymer-assisted scanning electron microscopy (SEM) approach for imaging stretched DNA molecules and associated protein signals on silicon wafers. Here, we present a practical protocol for implementing this workflow using λ DNA and streptavidin–fluorescent protein-labeled λ DNA as model DNA samples. The protocol encompasses silicon wafer preparation, microchannel-guided DNA deposition, heavy-metal staining with UranyLess, polyvinylpyrrolidone (PVP) treatment of the deposited DNA sample, SEM imaging, and image analysis. UranyLess provides heavy-metal staining to enhance SEM contrast, whereas the PVP concentration modulates the relative visibility of DNA backbones and protein-associated signals. Low-PVP conditions facilitate protein-signal visualization, while high-PVP conditions enhance DNA-backbone imaging. We also describe optional in-channel and droplet-based methods for delivering UranyLess and PVP. Finally, the protocol covers DNA backbone tracing, intensity-profile analysis, and machine-learning-based determination of protein positions from SEM images. Representative dCas9-bound DNA samples further demonstrate the applicability of the workflow to different protein-binding patterns, including closely spaced binding across a repetitive target array and localized binding on genomic DNA. This workflow provides a practical framework for preparing stretched DNA samples on silicon wafers and analyzing DNA backbones and associated protein signals by SEM.

## Introduction

Single-molecule visualization of DNA provides direct access to molecular length, conformation, local compaction, and molecule-to-molecule heterogeneity. When combined with protein detection, this type of imaging can further reveal where proteins bind along individual DNA molecules and how binding patterns vary between molecules. These positional readouts are difficult to obtain from ensemble biochemical assays, which average signals over many molecules and generally lose information about spatial organization along the DNA backbone. For this reason, imaging-based approaches have been widely used to study DNA structure, DNA–protein interactions, and molecular labeling along individual DNA molecules.

Fluorescence microscopy [1] is widely used for single-molecule DNA imaging because it is compatible with a wide range of molecular labeling strategies and microfluidic stretching, but optical diffraction limits the separation of closely spaced features. AFM [2–5] and TEM [6–8] provide higher spatial detail, yet these approaches can be limited by slow probe-based scanning, grid-based sample preparation, or practical difficulties in large-area searches for sparse molecules. SEM offers a complementary format because DNA samples can be prepared and imaged on rigid planar substrates such as silicon wafers, rather than on grid-based supports used for TEM. This planar substrate format allows large-area survey imaging and high-magnification observation to be performed on the same sample, while remaining compatible with surface functionalization and microchannel-based DNA deposition [9–13].

A major practical challenge is that DNA has weak intrinsic SEM contrast. DNA is composed mainly of low-atomic-number elements, and unstained DNA is therefore difficult to distinguish reliably from the silicon background. We recently established an UranyLess/PVP-assisted SEM approach to address this limitation and to visualize stretched DNA molecules and protein-bound regions on silicon wafers [14]. In this approach, UranyLess [15] provides heavy-metal staining components for SEM contrast, whereas polyvinylpyrrolidone (PVP) [16, 17] modulates the visibility of the DNA backbone and protein-associated signals. Using this strategy, protein-bound regions can be visualized as localized SEM signals along traced DNA backbones.

Here, we describe a practical protocol for UranyLess/PVP-assisted SEM imaging of stretched DNA and protein-bound DNA molecules. We focus on λ DNA and streptavidin–fluorescent protein-labeled λ DNA [18] as model DNA samples for DNA-backbone imaging and protein-position readout. The protocol covers silicon wafer preparation, microchannel-guided DNA deposition, UranyLess staining, PVP treatment of the deposited DNA sample, SEM imaging, and image analysis. We also describe how PVP concentration can be adjusted to emphasize either DNA-backbone visualization or protein-associated signals, and we summarize optional in-channel and droplet-based delivery formats for UranyLess and PVP. Finally, we outline DNA backbone tracing, intensity-profile analysis, and machine-learning-based protein-position readout from SEM images.

## Materials and methods

### Materials, reagents, and equipment

The key materials, reagents, and equipment used in this protocol are listed in Supplementary Table S1.

### Overview of the in-channel UranyLess/PVP workflow

**Figure 1:**
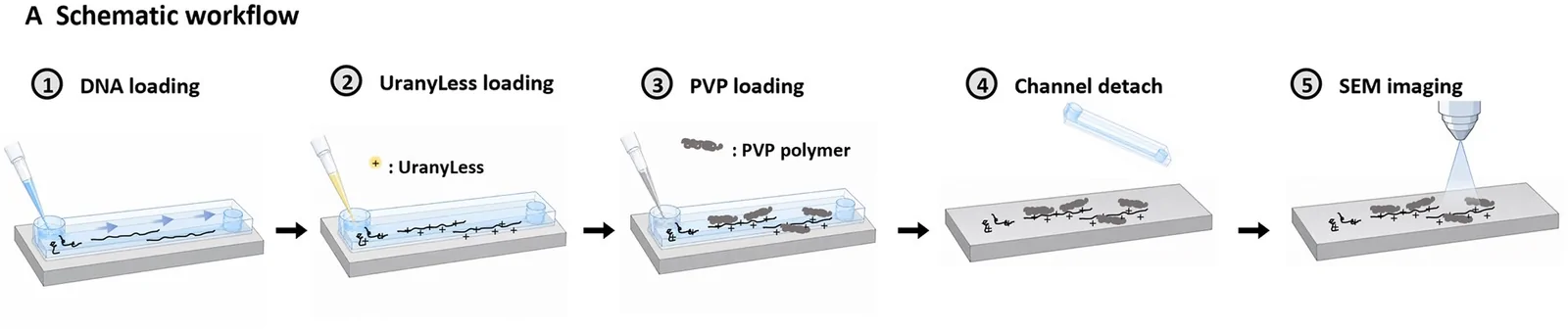
Overview of the in-channel UranyLess/PVP workflow for SEM-based DNA imaging. DNA molecules are loaded into a microchannel assembled on a silicon wafer to promote surface deposition and stretching. UranyLess is then introduced through the same channel to provide heavy-metal staining components, followed by PVP treatment to modulate SEM contrast. After sequential in-channel staining, the microchannel is detached, leaving the stained DNA molecules on the silicon wafer for SEM imaging.

We first describe the in-channel UranyLess/PVP workflow as a representative sample-preparation route for SEM imaging of stretched DNA molecules on silicon wafers. This workflow consists of five sequential steps: DNA loading, UranyLess staining, PVP treatment, microchannel detachment, and SEM imaging (Fig. 1). DNA molecules are introduced into a microchannel assembled on a silicon wafer, where capillary flow and surface adsorption promote deposition and stretching of individual molecules. UranyLess is then loaded through the same channel to provide heavy-metal staining components for SEM contrast, followed by PVP treatment to modulate the visibility of DNA backbones and protein-associated signals. The microchannel is then detached, leaving stained DNA molecules on the silicon wafer for SEM imaging. The following sections describe each step of this workflow in detail.

### Silicon wafer and microchannel setup

Commercial silicon wafers with a 30-nm SiO_2_ layer were purchased from Wafer Market (Yong-In, Korea) and used as substrates for DNA deposition and SEM imaging [14, 16]. The SiO_2_ surface is cleaned and silanized to generate a positively charged surface that promotes DNA adsorption. The procedure was adapted from a glass-coverslip preparation method for single-molecule DNA deposition [19].

*Note: Positively charged silicon wafers improve DNA adsorption, especially when DNA input is limited or when reproducible deposition is required.*

### Preparation of positively charged silicon wafers

1. Place silicon wafers with a 30-nm SiO_2_ layer in a PTFE holder.
2. Place the PTFE holder in a clean glass beaker and add piranha etching solution, prepared by mixing sulfuric acid and hydrogen peroxide at a 7:3 (v/v) ratio, until the wafers are fully submerged. Clean the wafers for 3 h without agitation.
*Caution: Piranha solution is highly corrosive and reacts violently with organic materials. Handle only in a chemical fume hood according to institutional safety procedures.*
3. Remove the piranha solution and rinse the wafers thoroughly with deionized water at least five times in the beaker.
*Waste disposal: Piranha solution waste should be collected and disposed of according to institutional hazardous-waste procedures. Do not mix piranha waste with organic solvents or organic waste.*
4. Sonicate the wafers in deionized water for 30 min.
*Optional: For additional cleaning, rinse the wafers twice with 99.9% ethanol after sonication.*
5. Rinse the wafers again thoroughly with deionized water at least twice to remove residual piranha solution.
6. Immediately before silanization, prepare 250 ml of silanization solution in a clean polypropylene jar by adding 100 µl of N-trimethoxysilylpropyl-N,N,N-trimethylammonium chloride in 50% methanol to deionized water.
*Note: Prepare the silanization solution immediately before use because N-trimethoxysilylpropyl-N, N, N-trimethylammonium chloride is sensitive to humidity.*
7. Transfer the PTFE holder containing the cleaned wafers to the silanization solution. Incubate the wafers at 65°C with agitation at 100 rpm for 16 h.
8. After incubation, discard the silanization solution and rinse the wafers three times with 99.9% ethanol in the same polypropylene jar.
9. Transfer only the silicon wafers to a clean conical tube containing 99.9% ethanol and store them in 99.9% ethanol until use.
10. Before DNA deposition, remove the wafer from ethanol and air-dry the surface.

### PDMS microchannel preparation

PDMS microchannels [20, 21] are used to guide DNA solution across the silicon wafer and promote the deposition and stretching of DNA molecules. PDMS devices can be fabricated in advance using standard soft lithography or prepared from an existing SU-8 mold. The PDMS microchannels used in this study were approximately 100 µm wide, 2.4 µm high, and 2 mm long.

*Note: The microchannel is used to promote DNA elongation for backbone-based positional analysis. Under the surface and microchannel conditions used in this protocol, λ DNA molecules selected for analysis typically reached approximately 91%–92% of their expected contour length. The degree of elongation can vary with DNA length and conformation, surface properties, and microchannel geometry. For applications that do not require microchannel-guided stretching, droplet-based implementation routes are described in* *Fig. 4*.

1. Prepare a SU-8 mold for the PDMS microfluidic device using standard soft lithography.
*Note: Detailed SU-8 mold fabrication is not essential for this protocol and can be performed using standard soft-lithography procedures. The critical requirement is that the final PDMS device forms stable conformal contact with the silicon wafer and allows controlled sample loading through the channel. The fabrication procedure for the microchannel mold used in this study has been described previously [20].*
2. Place the SU-8 mold in a clean Petri dish.
3. Mix SYLGARD™ 184 silicone elastomer base and curing agent at a 10:1 ratio.
4. Pour the PDMS mixture over the SU-8 mold until the patterned region is fully covered.
5. Cure the PDMS on the mold in a 65°C oven for at least 4 h.
6. Carefully peel the cured PDMS from the mold.
7. Cut the PDMS device to the desired size and punch inlet/outlet holes at the reservoir positions if needed.
8. Remove dust from the PDMS surface before plasma treatment.
9. Treat the PDMS device with air plasma to make the channel surface hydrophilic.
10. Store the plasma-treated PDMS device floating on deionized water in a clean Petri dish until use.
11. Use the PDMS device from the day after plasma treatment. Before assembly, remove the PDMS device from deionized water and air-dry the surface.
*Note: Avoid using the PDMS device immediately after plasma treatment. Freshly plasma-treated PDMS can produce excessively rapid liquid flow through the channel, which may reduce staining efficiency. In our hands, PDMS devices are typically used from the day after plasma treatment.*
12. Place the PDMS microchannel onto the silicon wafer so that the channel region makes conformal contact with the wafer surface.
*Note: The PDMS device does not need to be permanently bonded to the silicon wafer. Reversible contact is sufficient because the channel is detached after DNA deposition and staining.*

### In-channel DNA loading, UranyLess staining, and PVP treatment

DNA loading, UranyLess staining, and PVP treatment are performed sequentially through the same PDMS microchannel assembled on the silicon wafer. This in-channel workflow allows DNA deposition, heavy-metal staining, and PVP treatment to occur without removing the microchannel before staining is complete. DNA, UranyLess, and PVP were introduced sequentially by capillary flow without drying the channel between loading steps.

1. Place the dried PDMS microchannel onto the silicon wafer and confirm that the channel region forms conformal contact with the wafer surface.
2. Load 1 µl of DNA solution into the inlet reservoir of the PDMS microchannel.
*Note: A typical DNA concentration is 0.05 ng/µl for λ DNA. The DNA concentration can be adjusted depending on the desired molecule density.*
3. Allow the DNA solution to enter the channel by capillary flow and incubate for 5 min.
4. Load 0.5 µl of UranyLess into the inlet reservoir and allow it to flow into the channel.
*Note: UranyLess EM stain was used as supplied without further dilution unless otherwise indicated.*
5. Incubate for 5 min to stain the DNA molecules.
6. Load 0.5 µl of PVP solution at the selected concentration (typically 0.5% or 5% [w/v], depending on the imaging target) into the inlet reservoir and allow it to flow into the channel.
7. Incubate the sample for at least 10 min before channel detachment.
*Note: Incubation times of 10–50 min after PVP loading generally produced usable samples. However, excessive drying can make channel detachment difficult and may damage the prepared DNA sample. Detach the channel before the sample becomes completely dry.*
8. Carefully detach the PDMS microchannel from the silicon wafer.
9. Proceed to SEM imaging after the wafer surface is sufficiently dry.
*Note: The delivery order of UranyLess and PVP can be adapted for different sample-preparation routes. For example, PVP may be introduced before UranyLess delivery, or UranyLess may be applied as a droplet after channel detachment. Droplet-based UranyLess staining is described in* *Fig. 4. For quantitative comparisons, use the same delivery order and format across samples.*

### SEM imaging

SEM imaging is performed after channel detachment and drying of the silicon wafer surface. Samples are imaged directly on silicon wafers without additional conductive metal coating.

1. Mount the dried silicon wafer sample onto an SEM specimen stub.
2. Load the sample into the SEM chamber and image under high-vacuum conditions.
3. Acquire SEM images using a field-emission SEM equipped with a secondary electron detector.
4. Use an accelerating voltage of 10–30 kV.
5. First, survey the sample at low magnification to identify regions containing stretched DNA molecules.
6. Acquire high-magnification images of individual DNA molecules or protein-bound regions for downstream analysis.
7. Export the original SEM images in TIFF format for image analysis.
*Note: In our experiments, SEM images were acquired using an Apreo 2S HiVac microscope equipped with a secondary electron detector under high-vacuum Preparation of positively charged silicon wafers conditions without conductive metal coating. Representative images were typically acquired at an accelerating voltage of 10 kV, a working distance of 10 mm, a beam current of 0.4 nA, and a scan time of 7.9 s per frame.*

### PVP-dependent contrast modes for DNA and protein-signal visualization

**Figure 2:**
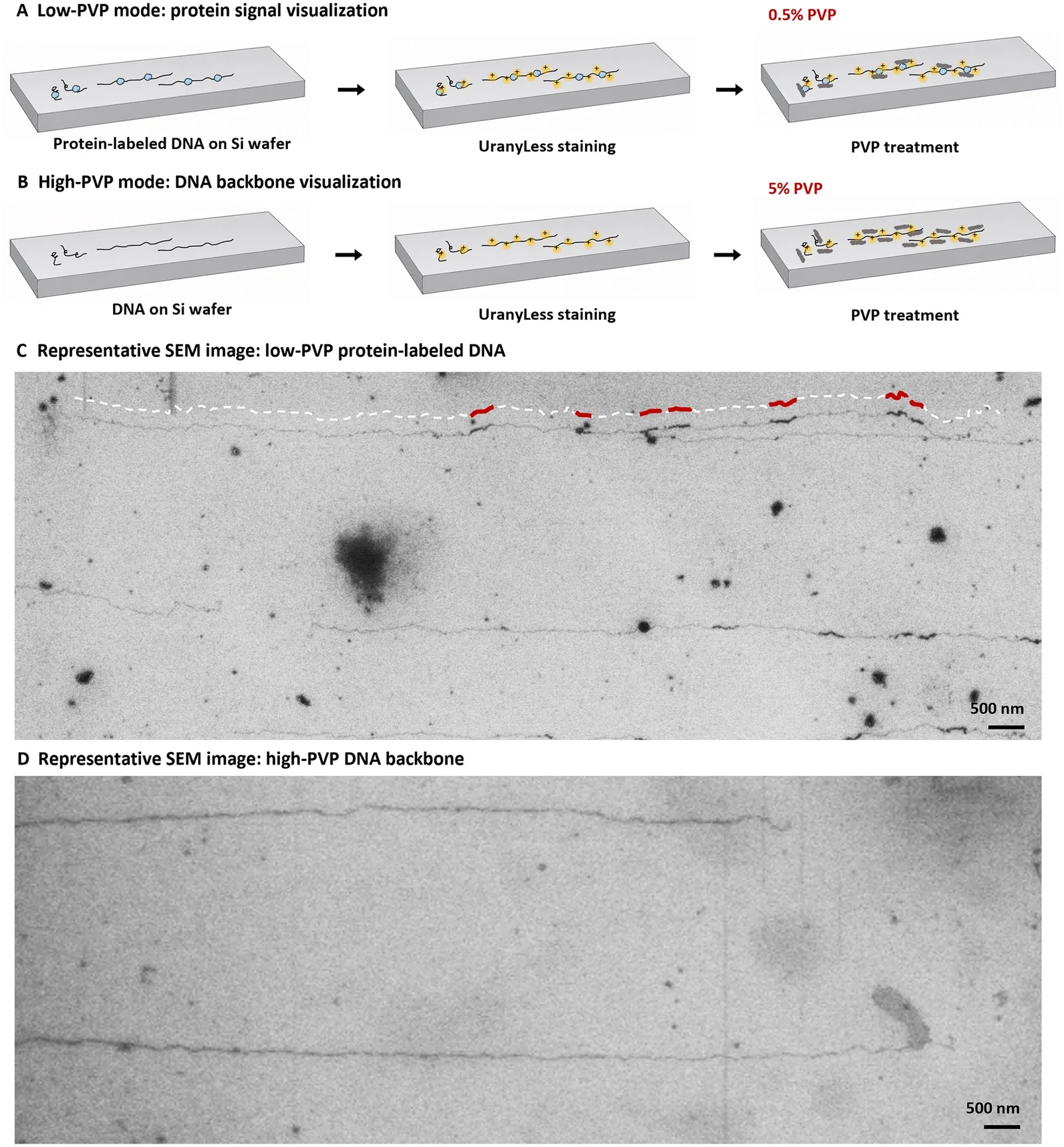
PVP-dependent contrast modes for protein-signal and DNA-backbone visualization. (A) Low-PVP mode for protein-signal visualization. Protein-bound DNA is deposited on a silicon wafer, stained with UranyLess, and treated with 0.5% PVP. This condition visualizes protein-associated signals while limiting excessive DNA-backbone enhancement. (B) High-PVP mode for DNA-backbone visualization. DNA molecules are stained with UranyLess and treated with 5% PVP to enhance DNA-backbone visibility. (C) Representative SEM image of SA-FP-labeled λ DNA prepared using the low-PVP mode. The traced DNA backbone is shown as a white dashed line, and detected protein-associated signals are highlighted in red. (D) Representative SEM image of unlabeled λ DNA prepared using the high-PVP mode, showing enhanced DNA-backbone contrast. Scale bars: 500 nm.

PVP concentrations are expressed as % (w/v). PVP solutions were prepared in deionized water using 40-kDa PVP, protected from light, stored at 4°C, and used within one month. Before use, the solutions were brought to room temperature and mixed thoroughly. Low-PVP conditions typically use 0.5% (w/v) PVP, whereas the high-PVP condition uses 5% (w/v) PVP. PVP concentration is adjusted according to the imaging target (Fig. 2). Low-PVP conditions are used when the goal is to visualize protein-associated signals while limiting excessive enhancement of the DNA backbone. In contrast, high-PVP conditions are used when the goal is to enhance DNA-backbone visibility. In both cases, UranyLess provides heavy-metal staining components for SEM contrast, whereas PVP modulates the apparent visibility of the DNA backbone and protein-associated signals. For protein-position readout, low-PVP conditions are used to provide sufficient DNA-backbone visibility for backbone tracing while retaining strong relative contrast of protein-associated signals; high-PVP imaging is a separate mode used primarily when enhanced visualization of the DNA backbone itself is desired.

1. For protein-signal visualization, prepare a low-PVP solution, typically 0.5% PVP.
*Note: PVP concentrations around 0.5%–1% can be tested depending on the sample condition and desired DNA-backbone visibility.*
2. After DNA loading and UranyLess staining, load 0.5 µl of low-PVP solution into the PDMS microchannel.
3. Incubate for at least 10 min before channel detachment.
4. For DNA-backbone imaging, prepare a high-PVP solution, typically 5% PVP.
5. After DNA loading and UranyLess staining, load 0.5 µl of high-PVP solution into the PDMS microchannel.
6. Incubate for at least 10 min before channel detachment.
*Expected outcome: Under low-PVP conditions, successful samples should show detectable localized protein-associated signals along traceable DNA molecules, while avoiding excessive darkening or thickening of the entire DNA backbone. Under high-PVP conditions, successful samples should show continuous DNA-backbone contrast with limited background particles and minimal DNA overlap.*
*Note: Low-PVP and high-PVP conditions should be selected according to the imaging purpose rather than treated as universally optimal conditions. Low-PVP conditions are useful for detecting localized protein-associated signals, whereas high-PVP conditions improve DNA-backbone visibility.*
*Note: Excessive PVP deposition or drying may increase background signal or make channel detachment difficult. If the channel becomes difficult to remove, reduce the incubation time after PVP loading or detach the channel before the sample becomes completely dry.*

### Preparation and SEM imaging of SA-FP-bound λ DNA

**Figure 3:**
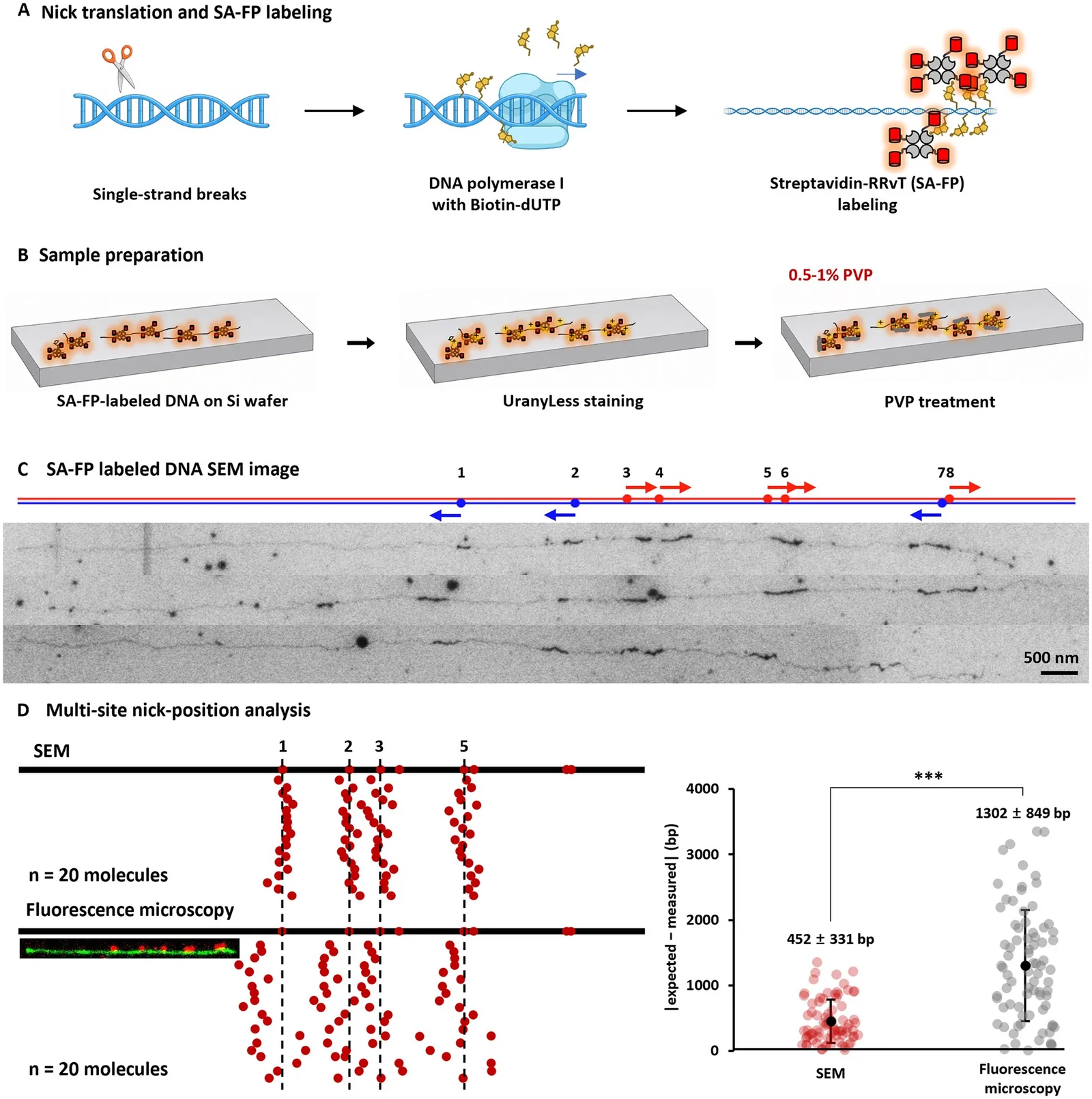
SEM-based positional readout of SA-FP-bound λ DNA using the low-PVP protocol. (A) Site-specific preparation of SA-FP-bound λ DNA. λ DNA is nicked to generate single-strand breaks, which are extended by DNA polymerase I in the presence of biotin-16-dUTP. Streptavidin–RRvT binding to the incorporated biotin generates SA-FP-bound regions on λ DNA. (B) Low-PVP sample preparation workflow for SA-FP-bound λ DNA. The DNA sample is deposited on a silicon wafer, stained with UranyLess, and treated with 0.5%–1% PVP to visualize protein-associated signals while limiting excessive DNA-backbone enhancement. (C) Representative SEM image of SA-FP-bound λ DNA prepared under the low-PVP condition. Expected nick-label positions are shown above the image, with red and blue arrows indicating the directions of DNA polymerase I-mediated nick translation on the two DNA strands. (D) Multi-site position readout by SEM and fluorescence microscopy. Detected SA-FP positions from 20 molecules are plotted relative to the expected nick sites. The smaller deviation from the expected positions in SEM supports backbone-based positional readout of SA-FP-bound λ DNA. Panel D was adapted from Noh *et al*. [14] under the CC by 4.0 license and expanded by adding five molecules to the previously reported dataset, resulting in *n* = 20 molecules per modality. Values are presented as mean ± SD. ***P < .001, Welch’s *t*-test.

SA-FP-bound λ DNA is used as a model protein-bound DNA sample for SEM-based protein-position readout (Fig. 3). In this protocol, we use streptavidin–RRvT (SA-RRvT), in which the tandem red fluorescent protein RRvT is fused to streptavidin [18]. RRvT was selected because its tandem fluorescent-protein architecture provides a relatively large protein label for SEM visualization while retaining fluorescence for comparison with fluorescence microscopy. Biotin-16-dUTP is incorporated into λ DNA by nick translation at Nb.BssSI nicking sites, followed by binding of SA-RRvT (hereafter referred to as SA-FP) to the incorporated biotin. The resulting SA-FP-bound λ DNA is then prepared using the low-PVP SEM workflow to visualize protein-associated signals along stretched λ DNA molecules.

### Streptavidin–RRvT fluorescent protein

Streptavidin–RRvT is a streptavidin-conjugated fluorescent protein in which RRvT is fused to the C-terminus of streptavidin through a GGSGG linker. The protein was expressed in *Escherichia coli* BL21(DE3) and purified by Ni-NTA affinity chromatography as previously described.

*Note: The complete amino acid sequence and detailed expression/purification procedure are provided in the Supplementary Methods. Purified streptavidin–RRvT is buffer exchanged into 1× TE buffer and stored at −20°C with glycerol.*

### Nick translation and SA-FP labeling of λ DNA

1. Prepare a nick-translation reaction mixture containing 500 ng of λ DNA, 20 units of Nb.BssSI, 10 units of DNA polymerase I, and 1× DNA Polymerase I Buffer.
2. Add the nucleotide mixture to achieve final concentrations of 1.1 µM biotin-16-dUTP and 5.5 µM each of dTTP, dATP, dCTP, and dGTP, and adjust the final reaction volume to 30 µl with nuclease-free water.
3. Incubate the reaction at 16°C for 2 h.
4. After nick translation, perform drop dialysis of the DNA solution against 1× TE buffer using a 25-nm mixed cellulose ester membrane for 2 h.
5. Recover the dialyzed DNA solution and dilute it to a final concentration of 2 ng/µl.
6. Mix the nick-translated DNA with streptavidin–RRvT at a final concentration of 150 nM.
7. Incubate the mixture at 20–25°C for 10 min to generate SA-FP-bound λ DNA.
8. Dilute the SA-FP-bound λ DNA to the working concentration used for SEM sample preparation.
*Note: For in-channel SEM preparation, DNA samples are typically loaded at 0.05 ng/µl. The concentration can be adjusted depending on molecule density and the frequency of overlapping DNA molecules.*

SA-FP-bound λ DNA is prepared for SEM imaging using the same low-PVP workflow described above; no additional modification of the SEM sample-preparation procedure is required. The steps are repeated below for completeness and to provide a self-contained protocol for this model protein-bound DNA.

### SEM preparation of SA-FP-bound λ DNA

1. Assemble the PDMS microchannel on the silicon wafer as described above.
2. Load 1 µl of SA-FP-bound λ DNA into the PDMS microchannel.
3. Incubate for 5 min to allow DNA deposition and stretching.
4. Load 0.5 µl of UranyLess into the channel.
5. Incubate for 5 min.
6. Load 0.5 µl of low-PVP solution, typically 0.5% PVP, into the channel.
7. Incubate for at least 10 min before channel detachment.
8. Detach the PDMS microchannel before the sample becomes completely dry.
9. Image the sample by SEM as described in the SEM imaging section.
*Note: Low-PVP conditions are used for SA-FP-bound λ DNA because they allow protein-associated signals to be visualized while limiting excessive DNA-backbone enhancement.*

### Optional implementation routes using PVP-preincubated DNA

**Figure 4:**
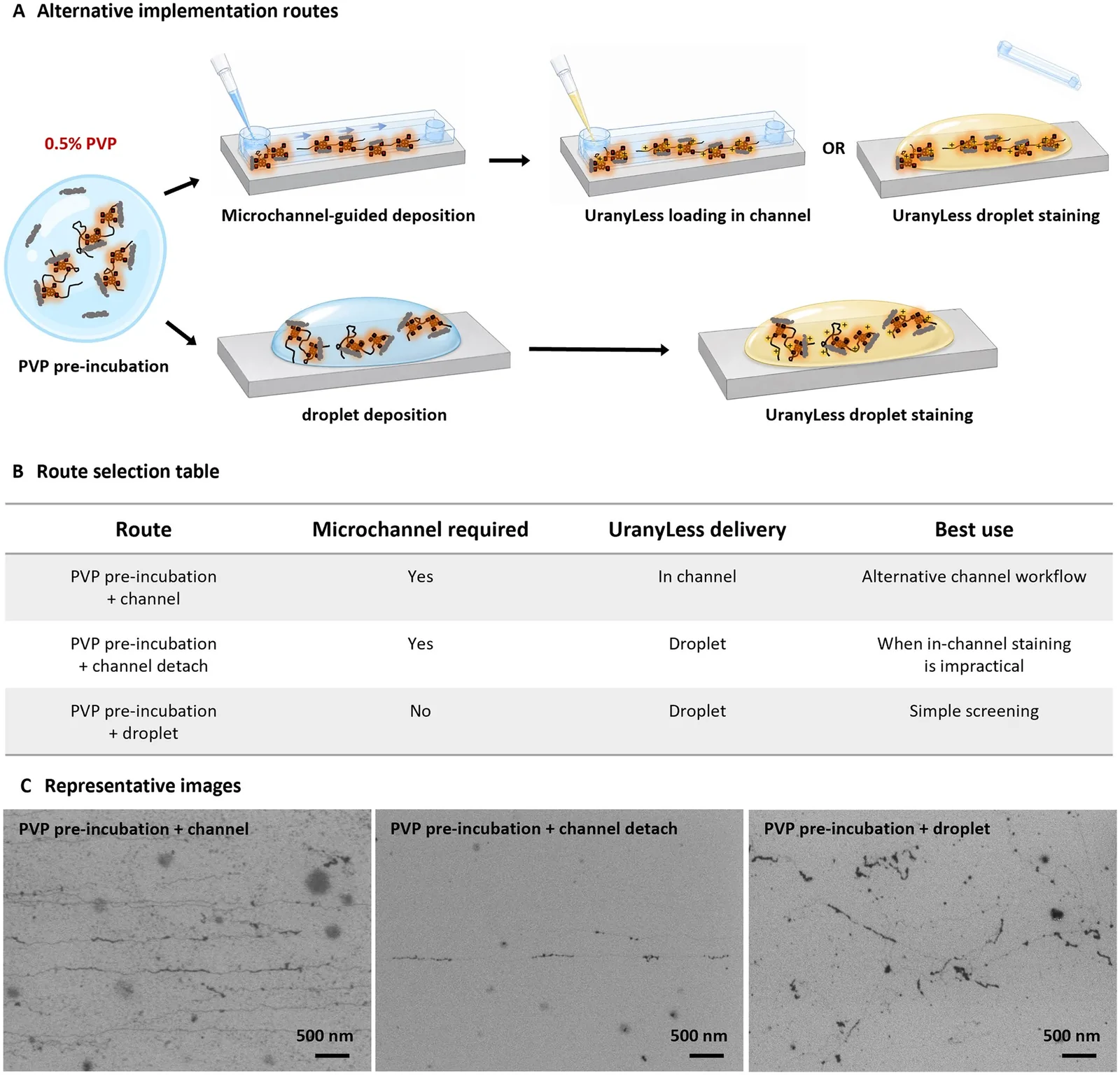
Optional implementation routes for UranyLess/PVP-assisted SEM sample preparation. (A) Schematic overview of alternative workflows using PVP-preincubated DNA samples. PVP-preincubated DNA can be deposited by microchannel-guided deposition or by direct droplet deposition. In the microchannel-guided route, UranyLess can be introduced through the channel or applied as a droplet after channel detachment. In the droplet-based route, UranyLess is applied directly as a droplet after deposition of the PVP-preincubated DNA sample. (B) Route selection table comparing the microchannel requirement, UranyLess delivery format, and recommended use for each optional workflow. (C) Representative SEM images obtained from the three optional preparation routes: PVP pre-incubation followed by microchannel-guided deposition with in-channel UranyLess delivery, PVP pre-incubation followed by microchannel-guided deposition with droplet-based UranyLess staining after channel detachment, and PVP pre-incubation followed by direct droplet deposition with droplet-based UranyLess staining. The images illustrate representative imaging outcomes for each preparation route.

PVP can also be introduced before DNA deposition by pre-incubating the DNA sample with PVP (Fig. 4). For the microchannel-guided routes (Routes A and B), λ DNA was prepared at 0.033 ng/µl with a final PVP concentration of 0.5% (w/v). For direct droplet deposition (Route C), λ DNA was prepared at 0.5 ng/µl with a final PVP concentration of 0.5% (w/v), and 2 µl of the DNA/PVP mixture was applied to the silicon wafer. The higher DNA concentration used for Route C was selected to obtain a suitable surface density of DNA molecules over the larger deposition area.

The two microchannel-guided routes differ primarily in the mode of UranyLess delivery. In Route A, UranyLess is introduced through the microchannel and generally produces stronger DNA-backbone contrast. Although this can improve backbone visibility, excessive DNA contrast can occasionally reduce the distinction between the DNA backbone and protein-associated signals. In Route B, the microchannel is detached after DNA deposition, and UranyLess is subsequently applied as a droplet. This route generally produces lower DNA-backbone contrast, which can facilitate discrimination of protein-associated signals, although UranyLess staining can occasionally be insufficient. In practice, Routes A and B can therefore be prepared in parallel, and the preparation providing the more suitable contrast can be selected for imaging. Route C provides a channel-free alternative for simpler sample handling when controlled DNA stretching is not required. The practical features of the three routes are summarized in Fig. 4B, and representative SEM images illustrating typical imaging outcomes are shown in Fig. 4C. Because the deposition format and DNA concentration differ among the routes, these representative images are intended to illustrate practical imaging outcomes rather than provide a quantitative comparison of performance.

1. Prepare the λ DNA/PVP mixture according to the selected deposition route: 0.033 ng/µl λ DNA with 0.5% (w/v) PVP for Routes A and B, or 0.5 ng/µl λ DNA with 0.5% (w/v) PVP for Route C.
2. Incubate the DNA/PVP mixture at room temperature for 5 min.
3. Proceed with one of the following deposition and UranyLess delivery routes.

### Route A: Microchannel-guided deposition with in-channel UranyLess staining

1. Assemble the PDMS microchannel on the silicon wafer.
2. Load 1 µl of the 0.033 ng/µl λ DNA mixture containing 0.5% (w/v) PVP into the PDMS microchannel.
3. Incubate for 5 min to allow DNA deposition and stretching.
4. Load 0.5 µl of UranyLess into the same microchannel.
5. Incubate for at least 10 min before channel detachment.
6. Detach the PDMS microchannel before SEM imaging.

### Route B: microchannel-guided deposition with droplet-based UranyLess staining

1. Assemble the PDMS microchannel on the silicon wafer.
2. Load 1 µl of the 0.033 ng/µl λ DNA mixture containing 0.5% (w/v) PVP into the PDMS microchannel.
3. Incubate for 5 min to allow DNA deposition and stretching.
4. Detach the PDMS microchannel.
5. Apply UranyLess directly onto the deposited DNA region as a droplet. Use a sufficient volume to cover the DNA-containing region; approximately 10 µl is typically sufficient.
6. Incubate for 5 min.
7. Remove the UranyLess solution by tilting the wafer and gently absorbing it with a Kimtech wipe.
8. Air-dry the wafer before SEM imaging.
*Note: Microchannel-guided deposition is useful when stretched DNA molecules are required for backbone-based readout. Direct droplet deposition can be used for simpler sample handling or rapid condition screening when molecular stretching is not essential.*

### Route C: direct droplet deposition with droplet-based UranyLess staining

1. Apply 2 µl of the 0.5 ng/µl λ DNA mixture containing 0.5% (w/v) PVP directly onto the silicon wafer as a droplet.
2. Invert the wafer and place it on a clean glass slide so that the droplet spreads across the wafer surface.
3. Incubate for 1 min.
4. Remove the wafer from the glass slide.
5. If liquid remains on the wafer surface, gently absorb the residual solution using a Kimtech wipe.
6. Apply UranyLess as a droplet to cover the deposited DNA region.
7. Incubate for 5 min.
8. Remove the UranyLess solution by tilting the wafer and gently absorbing it with a Kimtech wipe.
9. Air-dry the wafer before SEM imaging.
*Note: In droplet-based workflows, avoid disturbing the deposited DNA region when removing the droplet. Gently absorb liquid from the edge of the droplet rather than wiping across the wafer surface.*
*Note: UranyLess and PVP are the key contrast-generating components, and their delivery format can be adapted to the sample preparation route.*

### Image analysis workflow for SEM-based protein-position readout

DNA backbone tracing is used to convert the 2D trajectory of an individual DNA molecule in an SEM image into a 1D positional coordinate along the molecule. This step is important because deposited DNA molecules are not always perfectly linear and may exhibit local curvature even after stretching. Direct measurement along an image axis would therefore introduce positional errors, whereas backbone tracing preserves the actual path of the DNA molecule. Once the backbone is traced, local SEM intensity profiles can be sampled perpendicular to the DNA path at successive positions, allowing backbone-associated and protein-associated signals to be analyzed as a function of distance along the molecule. Thus, backbone tracing provides the spatial reference required for subsequent intensity-profile analysis and protein-position readout rather than serving primarily as an analysis of DNA morphology.

**Figure 5:**
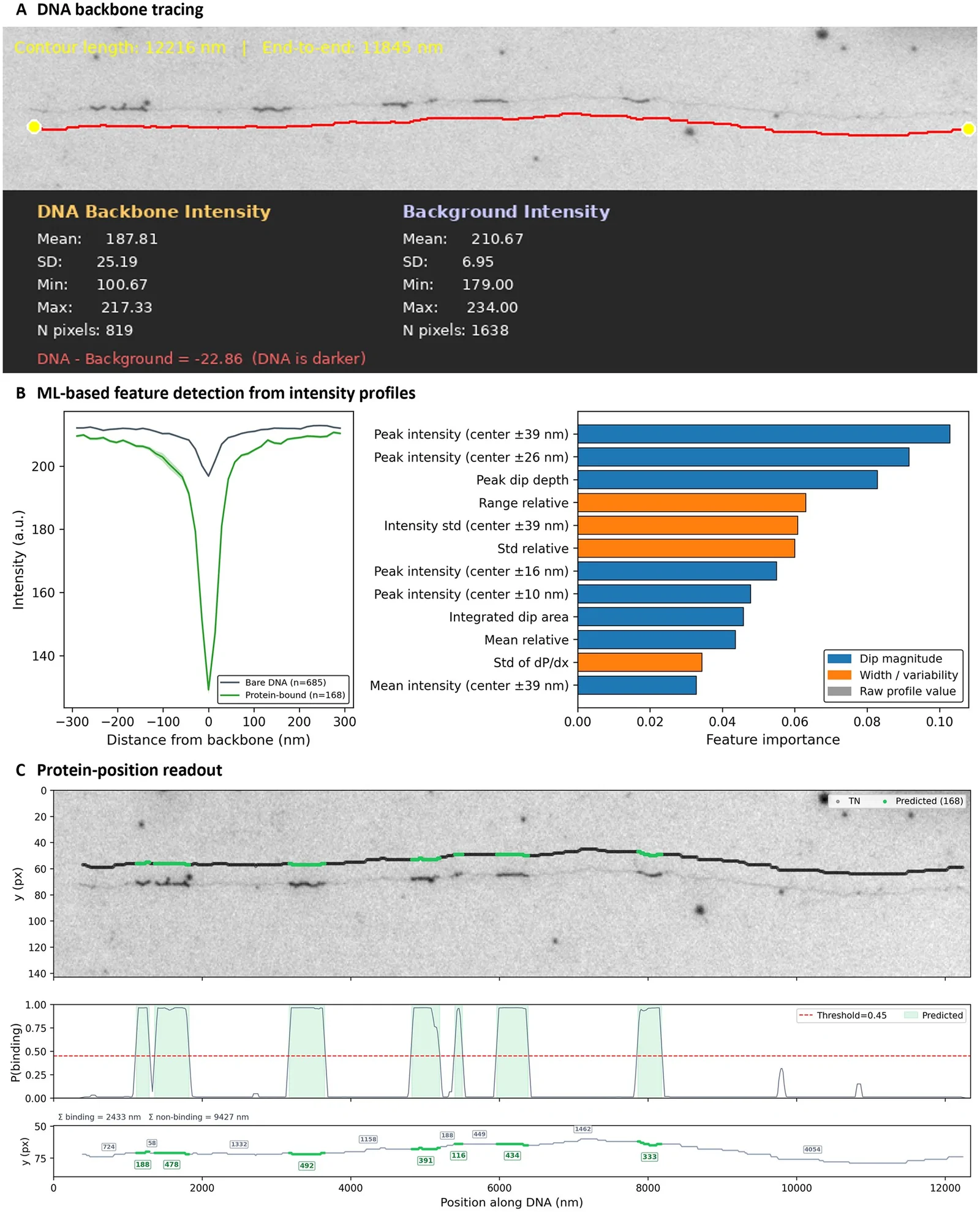
Image analysis workflow for SEM-based protein-position readout. (A) DNA backbone tracing and intensity extraction. A representative SEM image is used to trace the DNA backbone, thereby establishing a 1D positional coordinate along the molecule. Transverse intensity profiles are then extracted perpendicular to the traced backbone at successive positions to compare the local DNA-backbone signal with the adjacent background. (B) ML-based feature detection from intensity profiles, including representative transverse intensity profiles for bare and protein-bound DNA regions and relative importance of representative profile-derived features. (C) Protein-position readout. Predicted protein-bound regions are mapped onto the traced DNA backbone in the SEM image, and the corresponding binding probability profile and final segment-wise position readout are shown as a function of length along the DNA backbone.

SEM images were analyzed using a backbone-based image-analysis workflow to convert protein-associated SEM signals into positional readouts along individual DNA molecules (Fig. 5). The analysis pipeline starts from original TIFF images, traces DNA backbones [22–25], extracts transverse intensity profiles, and applies machine-learning-based classification [26–29] to identify protein-associated regions. Protein-associated regions are distinguished from the DNA backbone based on differences in the local transverse intensity profiles extracted along the traced backbone. The classifier uses profile-derived features describing local signal intensity and profile shape to identify regions that differ from the surrounding DNA-backbone signal. Detected regions are then mapped back onto the DNA backbone to generate probability profiles and segment-wise protein-position readouts. The analysis code, example files, and execution instructions are available on GitHub.

1. Prepare original SEM image files in TIFF format.
2. Configure the pixel scale for each image.
*Note: The analysis scripts require a per-image pixel scale in px/µm. If the image is not listed in the scale configuration file, the script can prompt for the value interactively.*
3. Trace the DNA backbone from the SEM image.
4. Measure the DNA edge-to-edge width and extract intensity information along the traced DNA backbone.
5. Generate perpendicular profile heatmaps from the traced DNA molecule.
6. Manually annotate protein-bound regions in the profile heatmap.
*Note: In the provided workflow, protein-bound rows in the profile heatmap spreadsheet are highlighted in yellow and used as ground-truth labels for machine-learning training.*
7. Train a supervised classifier using the labeled profile heatmaps.
8. Apply the trained classifier to new SEM images to predict protein-associated regions along traced DNA backbones.
9. Generate overlay images and prediction spreadsheets for visual inspection and quantitative analysis.
10. Convert predicted protein-associated regions into segment-wise positional readouts as a function of length along the DNA backbone.
*Note: The position coordinate is defined as the length along the traced DNA backbone rather than the straight-line distance between two points, because stretched DNA molecules can still contain local curvature.*
*Note: Predicted protein-associated regions should be visually inspected using the overlay images. False-positive signals can arise from contamination, background particles, DNA crossings, or locally uneven staining.*

## Results

**Figure 6:**
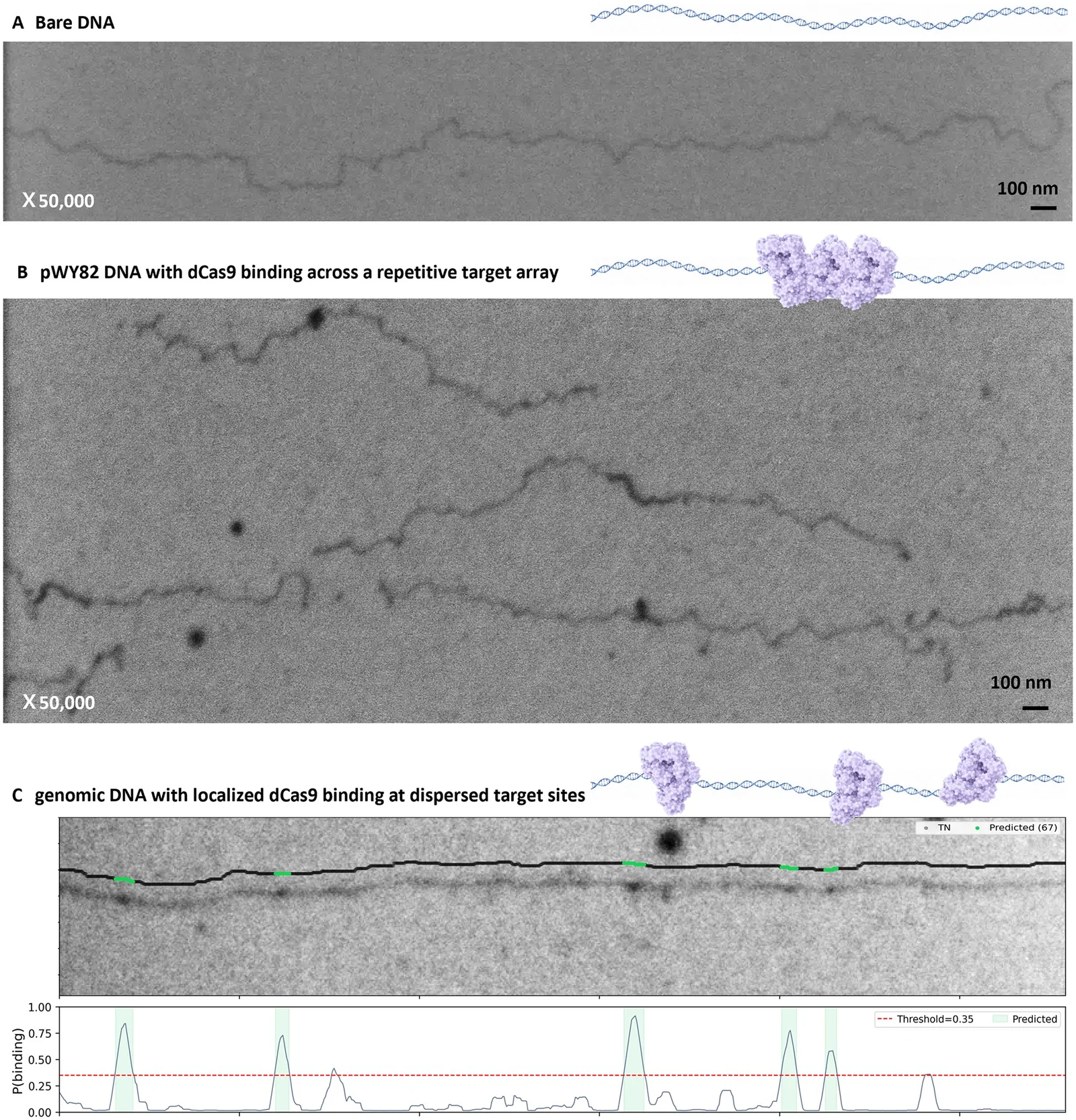
Representative SEM visualization and analysis of bare and dCas9-bound DNA molecules. (A) Representative SEM image of bare DNA, showing visible DNA-backbone contrast. (B) Representative SEM image of pWY82 plasmid DNA containing a repetitive telomeric target array, showing extended protein-associated SEM signals from multiple closely spaced dCas9-binding events. (C) Representative SEM image and analysis output of genomic DNA with localized dCas9 binding at dispersed sequence-specific target sites. The traced DNA backbone is shown in black, and predicted dCas9-associated regions are shown in green. The corresponding probability profile indicates predicted binding regions along the DNA backbone. Schematic illustrations above each SEM image indicate the corresponding bare or dCas9-bound DNA configuration. Scale bars in panels A and B: 100 nm.

In our previous work, we established UranyLess/PVP-assisted SEM imaging as a platform for visualizing stretched DNA molecules and protein-bound regions on silicon wafers [14]. In the present protocol, λ DNA and SA-FP-bound λ DNA are used as model DNA samples to describe the detailed sample-preparation and analysis steps. To illustrate how the same workflow can be extended to direct protein–DNA complexes, we also applied the protocol to dCas9-bound DNA molecules and examined representative SEM imaging outcomes (Fig. 6).

Bare DNA molecules appeared as continuous dark-line structures on the silicon wafer, allowing the DNA backbone to be followed at high magnification (Fig. 6A). When the protocol was applied to pWY82 plasmid DNA containing a repetitive telomeric target array that supports multiple closely spaced dCas9 binding events [30], extended protein-associated SEM signals were observed along DNA molecules (Fig. 6B). We further examined genomic DNA targeted at dispersed sequence-specific sites [31], where localized dCas9-associated signals appeared as discrete regions along the DNA backbone (Fig. 6C). Using the image-analysis workflow described above, the traced backbone and prediction probability profile provided a representative example of computational readout from localized protein-associated SEM signals. These examples show that the protocol can support visualization of bare DNA, multiple closely spaced protein-binding events, and localized protein-binding events, while maintaining compatibility with downstream backbone-based analysis.

## Discussion

This protocol describes a practical workflow for UranyLess/PVP-assisted SEM imaging of stretched DNA and protein-bound DNA molecules on silicon wafers. The method combines surface-based DNA deposition, heavy-metal staining, PVP-mediated contrast modulation, SEM imaging, and backbone-based image analysis. By organizing these steps into a reproducible sample-preparation and analysis workflow, the protocol provides an accessible route for laboratories that wish to apply SEM to single-molecule DNA visualization and protein-position readout.

A key feature of this workflow is the use of UranyLess and PVP as modular contrast-generating components. UranyLess provides heavy-metal staining components that enable SEM visualization of DNA on silicon wafers, whereas PVP modulates the apparent visibility of the DNA backbone and protein-associated signals. Adjusting the PVP condition allows the user to emphasize different imaging targets. Low-PVP conditions are useful for visualizing protein-associated SEM signals while limiting excessive DNA-backbone enhancement, whereas high-PVP conditions can be used when DNA-backbone visibility is the main goal.

The precise physicochemical mechanism underlying the PVP-dependent contrast modulation has not been fully established. Based on our previous observations [14], PVP likely associates more favorably with protein surfaces than with bare or UranyLess-coated DNA at lower to intermediate concentrations, resulting in preferential enhancement of protein-associated regions under low-PVP conditions. As the PVP concentration increases, this preferential association may become less dominant, allowing more extensive polymer association with the DNA or UranyLess-coated DNA backbone and thereby increasing backbone visibility. Thus, the low- and high-PVP imaging modes likely reflect concentration-dependent differences in the relative association of PVP with protein and DNA-containing regions rather than fundamentally different staining mechanisms. Because polymer adsorption and local coating thickness were not directly measured, this interpretation remains tentative, and PVP concentration should be regarded as an empirically optimized parameter for selecting the desired imaging contrast. This flexibility is useful because different experiments may require different balances between backbone contrast, protein-signal visibility, and background level.

The protocol is also compatible with multiple sample-handling formats. The in-channel workflow provides a convenient route for depositing and stretching DNA molecules, followed by sequential UranyLess staining and PVP treatment. Alternative routes using PVP-preincubated DNA and droplet-based UranyLess delivery can also be used, depending on the available setup and experimental purpose. These options are particularly useful when microchannel-guided stretching is not essential or when rapid screening of staining conditions is desired. However, the selected delivery route should be kept consistent within a given experiment, especially when comparing signal intensity, background level, or protein-associated signals across samples.

The backbone-based image-analysis workflow extends the protocol from visual inspection to protein-position readout. By tracing the DNA backbone, extracting transverse intensity profiles, and applying machine-learning-based classification, protein-associated SEM signals can be expressed as positions along a 1D molecular coordinate. For DNA molecules with known identity or sequence-specific positional landmarks, these positions can be compared with expected binding or labeling sites, as illustrated here by the defined Nb.BssSI labeling sites on λ DNA. In the absence of such references, the workflow can still provide single-molecule information on the relative distribution, spacing, clustering, and molecule-to-molecule heterogeneity of protein-associated signals, but it cannot assign individual signals to specific genomic loci. Accordingly, the method is most informative for known DNA substrates or when combined with sequence-specific labeling, DNA barcoding, or other positional landmarks. This analysis is applicable to different protein-binding patterns, provided that the protein-associated signal is distinguishable from the DNA backbone and background.

Several practical limitations should be considered. DNA deposition and stretching can be affected by surface condition, DNA concentration, channel handling, and drying state. Excessive drying before channel detachment may disturb the sample, while uneven UranyLess/PVP deposition can increase background signal. In addition, overlapping DNA molecules, discontinuous backbone contrast, dust particles, or uneven staining can lead to ambiguous backbone tracing or false-positive predictions. Therefore, sample quality and prediction overlays should be visually inspected before quantitative interpretation.

In summary, this protocol provides a practical guide for preparing, imaging, and analyzing stretched DNA and protein-bound DNA molecules by UranyLess/PVP-assisted SEM. The workflow supports DNA-backbone visualization, protein-associated signal detection, flexible sample preparation, and backbone-based computational readout on silicon wafers.

## Supplementary Material

bpag052_Supplementary_Data

## Data Availability

The SEM image-analysis code is available on GitHub (https://github.com/jolaboffice/SEM_DNA_Analysis) and archived in Zenodo under DOI: 10.5281/zenodo.20827106. The repository includes execution instructions and a bundled example dataset for verifying the analysis workflow. Additional raw image data supporting this protocol are available from the corresponding author upon reasonable request.
