# Supplementary material for: Metal- and polymer-assisted SEM imaging of protein-bound DNA molecules: a protocol
Source: Biol Methods Protoc. 2026 Sep 4;11(1):bpag052. doi: 10.1093/biomethods/bpag052 (PMC13613057; doi:10.1093/biomethods/bpag052)
Supplement: Supplementary file 1 [file bpag052_supplementary_data.docx]

**Metal- and polymer-assisted SEM imaging of protein-bound DNA molecules: a protocol**

Chanyoung Noh, Taebin Yun, Minseo Kang, Kyubong Jo*

Department of Chemistry, Sogang University; Seoul 04107, Korea

* To whom correspondence should be addressed. Email: [jokyubong@sogang.ac.kr](mailto:jokyubong@sogang.ac.kr)

**Supporting Information (SI)**

**Supplementary Table S1. Key materials, reagents, and equipment used in this protocol**

**Supplementary Table S1A. DNA substrates, proteins, and contrast reagents**

| **Category** | **Item** | **Supplier / source** | **Use in protocol** |
| --- | --- | --- | --- |
| DNA substrate | Bacteriophage λ DNA, Cat. No. N3011S | New England Biolabs | Model long DNA sample; preparation of SA-FP-bound λ DNA |
| DNA substrate | pWY82 plasmid DNA | Addgene, plasmid #65721 | DNA sample with multiple closely spaced dCas9-binding events |
| DNA substrate | HG002 genomic DNA | Prepared from HG002 (GM24385) cells | Genomic DNA sample with localized dCas9-binding events |
| Protein / complex | Streptavidin–RRvT fluorescent protein | In-house / previously described [1] | Binding to biotinylated λ DNA |
| Protein / complex | EnGen Spy dCas9, SNAP-tag, Cat. No. M0652S | New England Biolabs | Preparation of dCas9-bound DNA molecules |
| Staining / contrast | UranyLess EM stain, Cat. No. 22409 | Electron Microscopy Sciences | Heavy-metal staining reagent for SEM contrast |
| Staining / contrast | Polyvinylpyrrolidone, PVP, 40 kDa | Sigma-Aldrich | Modulation of DNA and protein-associated SEM contrast |

**Supplementary Table S1B. Surface preparation, microfluidics, imaging, and analysis**

| **Category** | **Item** | **Supplier / source** | **Use in protocol** |
| --- | --- | --- | --- |
| Surface substrate | Oxidized silicon wafer with 30-nm SiO₂ layer | Wafer Market | Substrate for DNA deposition and SEM imaging |
| Surface reagent | N-Trimethoxysilylpropyl-N,N,N-trimethylammonium chloride | Gelest | Preparation of positively charged silicon wafer surface |
| Microfluidics | SYLGARD 184 silicone elastomer kit | Dow Corning / supplied through K1 Solution | PDMS microchannel fabrication |
| Dialysis | Mixed cellulose ester membrane, 25 nm | Merck | Drop dialysis after nick translation |
| Imaging equipment | Apreo 2S HiVac field-emission SEM | Thermo Fisher Scientific | SEM imaging |
| Analysis | SEM_DNA_Analysis code | GitHub / Zenodo | DNA backbone tracing and protein-position readout |

Polyvinylpyrrolidone (PVP, 40 kDa) was purchased from Sigma-Aldrich (St. Louis, MO, USA). Oxidized silicon wafers were purchased from Wafer Market (Yong-In, Korea). N-Trimethoxysilylpropyl-N,N,N-trimethylammonium chloride was obtained from Gelest (Morrisville, PA, USA). Bacteriophage λ DNA, Nb.BssSI, Proteinase K, β-Agarase I, and EnGen Spy dCas9 (SNAP-tag) were purchased from New England Biolabs (Ipswich, MA, USA). pWY82 plasmid DNA was obtained from Addgene (plasmid #65721). DNA polymerase I was obtained from Enzynomics (Daejeon, Korea). Biotin-16-dUTP was purchased from Jena Biosciences (Thuringia, Germany), and dNTPs were obtained from New England Biolabs. UranyLess EM stain was obtained from Electron Microscopy Sciences (Hatfield, PA, USA). Alt-R CRISPR-Cas9 tracrRNA (Atto 647-labeled), Alt-R CRISPR-Cas9 crRNA, and nuclease-free duplex buffer were obtained from Integrated DNA Technologies (Coralville, IA, USA). RPMI 1640 medium and 1× antibiotic–antimycotic were purchased from Gibco (Waltham, MA, USA). SYLGARD 184 silicone elastomer kit was purchased from Dow Corning and supplied through K1 Solution (Korea). The 25-nm mixed cellulose ester membrane was obtained from Merck. The Apreo 2S HiVac field-emission SEM was from Thermo Fisher Scientific.

**Supplementary Methods**

1. **Expression and purification of streptavidin–RRvT fluorescent protein**

Streptavidin–RRvT (SA-RRvT; hereafter referred to as SA-FP) was used for binding to biotinylated λ DNA. The SA-FP expression construct was prepared as previously described [1]. In this construct, the RRvT fluorescent protein is fused to the C-terminus of streptavidin through a flexible GGSGG linker, yielding a fusion protein with an expected molecular weight of approximately 73.5 kDa. The complete amino acid sequence of SA-FP is provided below.

MGSSHHHHHHSSGLVPRGSMASMTGGQQMGAGITGTWYNQLGSTFIVTAGADGALTGTYESAVGNAESRYVLTGRYDSAPATDGSGTALGWTVAWKNNYRNAHSATTWSGQYVGGAEARINTQWLLTSGTTEANAWKSTLVGHDTFTKVKPSAASIDAAKKAGVNNGNPLDAVQQ**GGSGG**PGMVSKGEEVIKEFMRFKVRMEGSMNGHEFEIEGEGEGRPYEGTQTAKLKVTKGGPLPFAWDILSPQFMYGSKAYVKHPADIPDYKKLSFPEGFKWERVMNFEDGGLVTVTQDSSLQDGTLIYNVKMRGTNFPPDGPVMQKKTMGWEASTERLYPRDGVLKGEIHQALKLKDGGHYLVEFKTIYMAKKPVQLPGYYYVDTKLDITSHNEDYTIVEQYERSEGRHHLFLYGMDELYKGSTGSGSSGPMVSKGEEAIKEFMRFKVSMEGSMNGHEFEIEGEGEGRPYEGTQTAKLKVTKGGPLPFAWDILSPQFMYGSKAYVKHPADIPDYKKLSFPEGFRWERVMNFEDGGLVTVTQDSSIQDGTLIYKVKVRGTNFPPDGPVMQKKTMGWEASTERLYPRDGVLKGEIHQALKLKDGGHYLVEFKTIYMAKKPVQLPGYYYVDTKLDITSHNEDYTVVEQYERSEGRHHLFLYGMDELYKGSGC

The SA-FP expression plasmid was transformed into *E. coli* BL21(DE3) cells using a standard transformation procedure. A single transformed colony was inoculated into Luria–Bertani medium containing ampicillin and cultured for 16 h at 37°C. The next day, 1 mL of the overnight culture was transferred to 100 mL of fresh Luria–Bertani medium containing ampicillin and incubated at 37°C until the optical density at 600 nm reached 0.4–0.6. Protein expression was induced by adding IPTG to a final concentration of 1 mM. The culture was then incubated overnight for 16 h at 20–25°C with shaking at 200 rpm. Cells were collected and disrupted by ultrasonication for 15 min, followed by centrifugation at 10,000 rpm for 10 min to remove cell debris. SA-FP was purified from the clarified lysate by Ni-NTA affinity chromatography. The bound protein was eluted using a buffer containing 50 mM Na₂HPO₄, 300 mM NaCl, and 250 mM imidazole at pH 8.0. The purified protein was buffer exchanged into 1× TE buffer consisting of 10 mM Tris and 1 mM EDTA at pH 8.0. Glycerol was added for storage, and the purified SA-FP protein was stored at −20°C until use.

2. **HG002 genomic DNA preparation**

HG002 (GM24385) cells were obtained from the Coriell Institute and used for genomic DNA preparation. Cells were cultured in RPMI 1640 medium supplemented with 15% fetal bovine serum and 1× antibiotic–antimycotic. For genomic DNA preparation, cells were harvested from the suspension culture by centrifugation at 1,000 × g for 5 min in a 15-mL conical tube. After removal of the culture medium, the cell pellet was resuspended in 1 mL of 1× PBS and transferred to a microcentrifuge tube. The cells were washed twice with 1× PBS and finally resuspended in 500 μL of 1× PBS. Genomic DNA was extracted using agarose gel plugs. The cell suspension was mixed with 2% low-gelling-temperature agarose prepared in 1× TE buffer to obtain a final agarose concentration of 0.7%. The mixture was divided into 20-μL aliquots and incubated at 4°C for 1 h to allow plug formation. Proteinase K was then added to a final concentration of 2 mg/mL, and the plugs were incubated at 42°C for 4 h. An additional equal amount of Proteinase K was added, followed by overnight incubation at 42°C for 16 h. After protein digestion, the agarose plugs were washed four times with 1× TE buffer for 30 min each. The plugs were then melted at 65°C for 20 min, and 1 μL of β-Agarase I was added to digest the agarose. The mixture was incubated at 42°C for 1 h, followed by heat inactivation of β-Agarase I at 65°C for 20 min. The resulting genomic DNA solution was used for downstream dCas9 binding and SEM sample preparation.

3. **Preparation of dCas9-bound pWY82**

Guide RNAs were prepared by annealing Atto 647-labeled tracrRNA with crRNA targeting the telomeric repeat sequence in pWY82 DNA. For gRNA annealing, 1 μL of 100 μM Atto647-labeled tracrRNA, 1 μL of 100 μM crRNA targeting GGTTTAGGGTTTAGGGTTTA, 1.6 μL of DEPC-treated water, and 0.4 μL of nuclease-free duplex buffer were mixed. The mixture was incubated at 95°C for 5 min and then cooled at 25°C for 10 min to allow annealing. The annealed gRNA was diluted with 96 μL of DEPC-treated water to obtain a final concentration of 1 μM. For dCas9 ribonucleoprotein assembly, equal volumes of 1 μM gRNA and 1 μM SNAP-tagged dCas9 were mixed and incubated at room temperature for 10 min. The dCas9-binding reaction was then prepared in a total volume of 20 μL by combining 50 ng of pWY82 plasmid DNA, 6.3 μL of gRNA–dCas9 ribonucleoprotein complex, 2 μL of 10× NEBuffer 3.1, and DEPC-treated water. The reaction mixture was incubated at 37°C for 1 h to generate dCas9-bound pWY82 DNA. The resulting DNA–dCas9 complex was used as a model sample containing multiple closely spaced dCas9-binding events for SEM sample preparation.

4. **Preparation of dCas9-bound HG002 genomic DNA**

dCas9-bound HG002 genomic DNA was prepared using two crRNAs targeting repetitive genomic sequences:

crRNA1: CTGCACTCCAGCCTGGGCGA

crRNA2: CACTTGAACCCAGGAGGCAG

Each crRNA was annealed with Atto 647-labeled tracrRNA using the same annealing procedure described above for pWY82 DNA. After annealing, each gRNA was diluted with 96 μL of DEPC-treated water to obtain a final concentration of 1 μM. Before ribonucleoprotein assembly, the two gRNAs were mixed at a molar ratio of 7:1. Equal volumes of the mixed gRNA solution and 1 μM SNAP-tagged dCas9 were then combined and incubated at room temperature for 10 min to form the gRNA–dCas9 ribonucleoprotein complex. DNA binding was performed in a total volume of 10 μL containing 5 ng of HG002 genomic DNA, 2.5 μL of gRNA–dCas9 ribonucleoprotein complex, 1 μL of 10× NEBuffer 3.1, and DEPC-treated water. The reaction was incubated at 37°C for 1 h to generate dCas9-bound HG002 genomic DNA. The resulting dCas9-bound genomic DNA sample was used as a model sample with localized dCas9-binding events for SEM sample preparation and image-analysis demonstration.

5. **Detailed image-analysis parameters**

DNA backbones were traced from SEM images using the SEM_DNA_Analysis workflow. After image enhancement, DNA-like line features were traced using a dynamic-programming-based algorithm. Transverse intensity profiles were extracted perpendicular to the local tangent direction along each traced DNA molecule and resampled onto a common nanometer-scale grid. Protein-associated SEM signals were detected using supervised classification of the extracted transverse profiles. Manually annotated profile heatmaps were used to define protein-associated and non-protein-associated regions, and profile-derived features were used to train a classifier. The classifier generated prediction probabilities along the DNA backbone, which were further processed using smoothing along the one-dimensional backbone coordinate, thresholding, and a minimum run-length filter to identify predicted protein-associated regions. Because the annotated profile number, class balance, trained classifier, and optimized thresholds depend on the image dataset and imaging conditions, users should validate or re-optimize the classifier when applying the workflow to new samples or SEM settings. The analysis scripts and representative trained model used in this study are available with the SEM_DNA_Analysis code.

1.	Jin Y, Bae J, Kim TY et al. Twelve Colors of Streptavidin-Fluorescent Proteins (SA-FPs): A Versatile Tool to Visualize Genetic Information in Single-Molecule DNA. Anal Chem 2022;94:16927-35.
